# Pareto-principle in rare disease education: assessing the representation of common rare diseases in medical education and coding systems

**DOI:** 10.1186/s13023-024-03347-y

**Published:** 2024-09-12

**Authors:** Alexandra Berger, Kai Lars Grimm, Richard Noll, Thomas OF Wagner

**Affiliations:** 1 Frankfurt Reference Centre for Rare Diseases, Goethe University Frankfurt, University Hospital, Theodor-Stern-Kai 7, 60590 Frankfurt am Main, Germany; 2 Institute of Medical Informatics (IMI), Goethe University Frankfurt, University Hospital, Theodor Stern Kai 7, 60590 Frankfurt am Main, Germany

**Keywords:** Most prevalent rare diseases, Medical education, ICD-10 coding

## Abstract

**Background:**

The Pareto Principle asserts that a large portion of results can be achieved with a small amount of effort. Wakap et al. found that around 80% of individuals with rare diseases (RD) suffer from one of 149 specific rare diseases. A significant challenge in the RD domain is the lack of information, compounded by the fact that most RD are not specifically codifiable in the ICD-10, leading to a deficit in reliable epidemiological data. Additionally, time constraints in medical education hinder the comprehensive teaching of all RD, contributing to the diagnostic odyssey problem through failure of recognizing diseases. We identified the most and second most prevalent RD (prevalences of 1–5/10,000 and 1–9/100,000, respectively) from the Orphanet Epidemiology File, totaling 454 diseases. We investigated the feasibility of specific coding using ICD-10-GM and whether these diseases were explicitly listed in the subject catalog (GK) of the second state examination in human medicine in Germany. A two-sided chi-square test was employed to identify statistically significant differences between prevalence groups.

**Results:**

Out of 454 diseases, a total of 34% could be specifically coded in ICD-10-GM, with 49% of diseases in the 1–5/10,000 prevalence range (153 RD) and 26% in the 1–9/100,000 range (301 RD) having specific codes. Approximately 15% of all investigated diseases were part of the GK, with 25% of the most prevalent and 10% of the second most prevalent RD group, respectively. Statistically significant differences were observed between prevalence groups concerning the presence of a specific ICD-10-GM code and inclusion in the GK.

**Conclusion:**

Only 49% of the most prevalent RD can be specifically coded, highlighting the challenge of limited epidemiological data on RD. In Germany, the Alpha-ID was introduced in addition to ICD-10 in the inpatient setting to obtain more valid epidemiological data on RD. Recognizing the Pareto Principle’s applicability, the study emphasizes the importance of including the most common rare diseases in medical education. While recognizing the limitations, especially in covering ultra-rare diseases, the study underscores the potential benefits of enhancing medical curricula to improve rare disease awareness and diagnostic accuracy.

**Supplementary Information:**

The online version contains supplementary material available at 10.1186/s13023-024-03347-y.

## Background

In the European Union, a rare disease is defined as a disease affecting less than 5 out of 10,000 people. An estimated 4–5 million people in Germany and 30 million people in Europe live with a rare disease, according to the Council of the European Union [1]. A major problem with rare diseases is a general lack of awareness and information [2, 3]. We want to focus on two fields where this information gap becomes apparent: The representation of rare diseases in the International Classification of Diseases (ICD) and in medical education.

### ICD-10

In the International Classification of Diseases version for Germany, ICD-10-GM, most rare diseases do not have a specific code. Aymé et al. showed, that only 517 out of 6,974 rare diseases (7,4%) can be mapped to a unique code or a set of codes in the ICD 10 WHO version [4]. This poses challenges in generating reliable information on disease prevalence and other epidemiological data, such as the total number of affected individuals, let alone assessing economic and social effects of rare diseases [3]. 

### Medical education

Undergraduate medical education shows significant differences among the European Union although there are many attempts to standardise the curricula. Despite learning objectives becoming more and more similar, the examinations still differ. There are member states like France, Italy, the Netherlands and Greece where central examinations are unknown. In Germany all students must undergo state exams [6–9]. The German state examinations are based on the national learning objectives (subject catalogue) of the Institute for Medical and Pharmaceutical Examination Questions (IMPP). This catalogue provides a comprehensive but not exhaustive overview of topics and subjects covered in medical and pharmaceutical examinations. It serves as a guideline for exam preparation and outlines the knowledge and competencies expected from medical and pharmaceutical graduates [10]. To our knowledge, there is no comparable catalogue in the EU. In Switzerland, where medical students must also take state exams, the PROFILES catalogue defines what is expected of medical graduates. PROFILES consists of professional activities such as history-taking and clinical situations, for example a patient presenting with abdominal pain, rather than specified diseases [11, 12]. In contrast, the German subject catalogue currently includes about 800 diseases or groups of diseases [10]. Given that there are about 8,000 known rare diseases, there is an obvious discrepancy between the number of rare diseases and the number of diseases taught to medical students. This may contribute to the diagnostic odyssey problem for people with rare diseases [13]. Doctors, who are unfamiliar with a particular rare or common disease, may struggle to reach an accurate diagnosis. Every individual, however, has equal rights to a correct diagnosis and treatment irrespective of the rarity of their illness.

Addressing this dilemma is a complex task. Expanding the subject catalogue for medical schools to include all rare diseases would require prolonging the standard period of study.

### Pareto principle

Vilfredo Pareto, a 19th century Italian economist, observed a pattern of “predictable imbalance” where 80% of Italy’s wealth was held by 20% of the population. It is often referred to as the “80/20 rule” as researchers began to observe similar phenomena in most systems that have inputs and outputs, including production and financial management. However, the 80/20 ratio should not be taken literally. The Pareto Principle merely indicates that the majority of outputs are often derived from a minority of inputs [14]. 

Wakap et al. showed, that the Pareto principle can be applied to the field of rare diseases. They analysed the Orphanet Epidemiology File to estimate the cumulative point prevalence of all rare diseases. As one of their results, they estimated that 3.5 to 5.9% of the global population is affected by a rare disease, making rare diseases a relevant topic for every healthcare professional. However, not all rare diseases are equally rare. Prevalence ranges from 1 in 2,000 affected individuals to isolated cases. Most rare diseases, though, have an extremely low prevalence, with fewer than 1 patient per 1 million people. Nevertheless, most patients with a rare disease suffer from a relatively more common rare disease. Wakap et al. concluded that approximately 80% of patients have one of 149 rare diseases. Moreover, 390 rare diseases account for up to 98% of all rare disease cases [15]. Knowledge of these common rare diseases may also be crucial for medical doctors, as the likelihood of encountering them during their careers is not negligible.

The aim of this study is to identify these common rare diseases, to examine whether they have a specific ICD-10-GM code and to assess their inclusion as mandatory components in the medical curriculum according to the IMPP subject catalogue. We want to prove the hypothesis, that the likelihood of having a specific ICD-10-GM code and the likelihood of being included in the subject catalogue depends on the prevalence of a rare disease.

## Methodology

We obtained the Orphanet Epidemiology File version of June 14, 2022 in September 2022. This file contains epidemiological data on 6,056 rare diseases [16]. To prevent duplication, we excluded subgroups and overarching disease groups (such as lysosomal storage disorders) from our analysis. This resulted in a total of 5,152 diseases.

We only included diseases with available data on point prevalence. Prevalence data is provided for specific geographic regions, including worldwide, across Europe, or individual countries. We excluded data for “specific populations“. For example, due to founder effects or selective advantages, there may be an increased occurrence of a particular disease in a specific population. This clustering of disease is not applicable to general population groups, which is why we removed this group from the analysis. Orphanet provides data on prevalence ranges in the categories > 1:1,000, 6–9/10,000, 1–5/10,000, 1–9/100,000, 1–9/1,000,000, < 1:1,000,000, unknown and not yet documented. We excluded the last two categories, as they do not provide any information, resulting in 4803 data elements for 3699 rare diseases. The distribution of rare diseases across the prevalence ranges is shown in Table 1. Multiple data elements may exist for a single disease, ranging from 1 to 109 data elements (109 being the maximum for haemophilia A). Therefore, one disease can be found in more than one prevalence range category. For our analysis, we prioritized the prevalence data in the following order: Germany, Europe, worldwide, and a single country with the highest prevalence. Considering, that diagnoses may be overlooked due to the rarity of the disease and the lack of specific coding options for rare diseases, we assumed that prevalence data are more likely to be underestimated than overestimated.

**Table 1:** Prevalence data of the 3,699 rare diseases, with some rare diseases having multiple data

Prevalence range	Number of rare diseases
> 1:1,000 and 6–9/10,000	24
1–5/10,000	196
1–9/100,000	306
1–9/1,000,000	268
< 1:1,000,000	3,024
∑	3,818

**Fig. 1:**
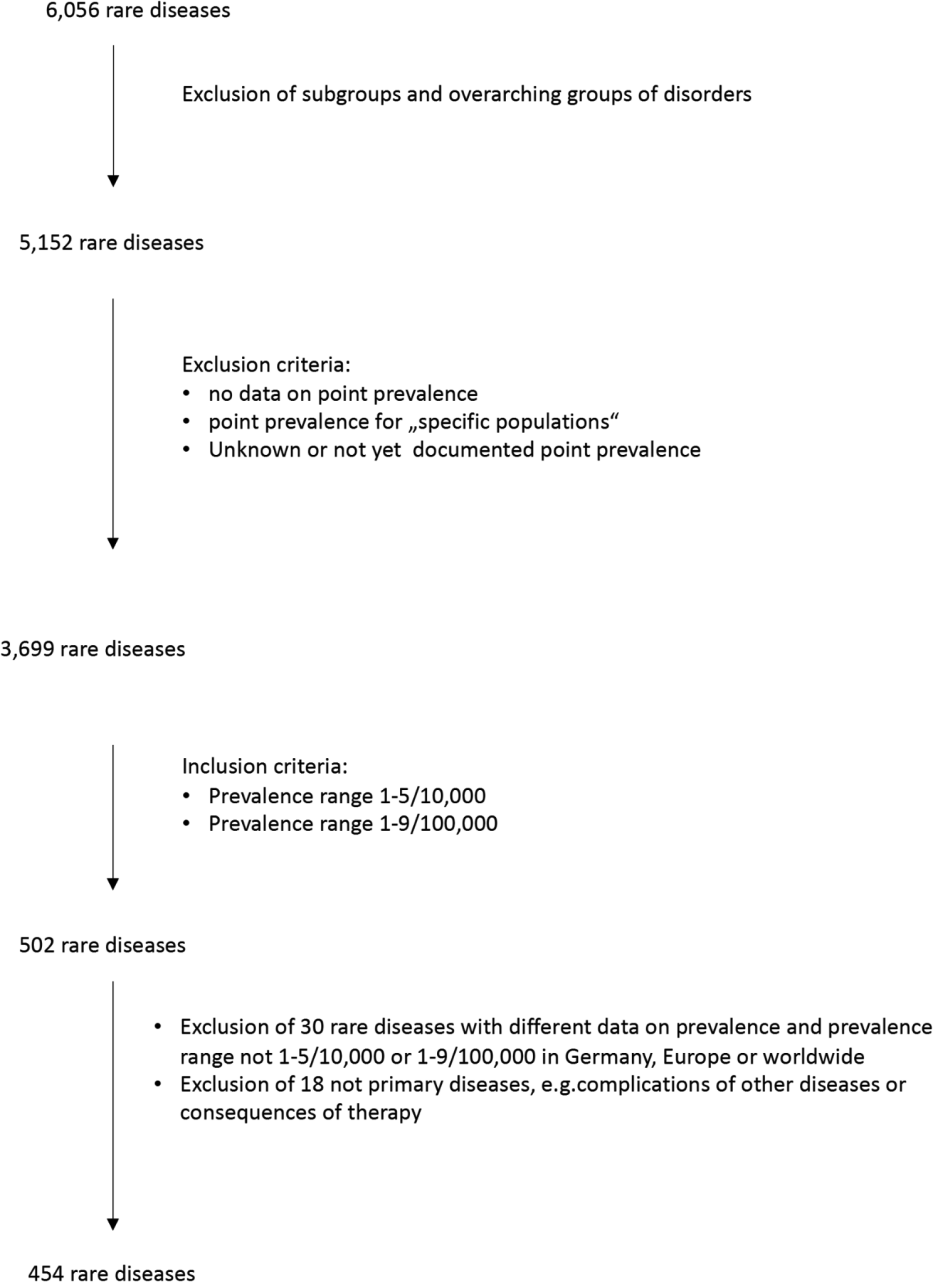
Inclusion/exclusion chart

In this paper, we define diseases within a prevalence range of 1–5/10,000 as the most prevalent rare diseases and those within a prevalence range of 1–9/100,000 as the second most prevalent rare diseases. Common rare diseases are defined as rare diseases within both of these prevalence groups. A rare disease with a prevalence of 9/1,000,000 or less is defined as ultra-rare in this paper. Our analysis focused on the common rare diseases, with target prevalence ranges of 1–5/10,000 and 1–9/100,000. We excluded 30 diseases from our analysis due to multiple prevalence data of which the prevalence range did not fall within the target range in Germany, Europe or worldwide. (Appendix: Table 4) Additionally, we had to exclude 18 entries describing complications or therapy consequences rather than primary disease entities (Appendix: Table 5). In total, we included 454 rare diseases in our analysis: 153 rare diseases with a prevalence of 1–5/10,000 and 301 rare diseases with a prevalence of 1–9/100,000. (Fig. 1). Analysis was performed separately for all 454 diseases, the 153 and the 301 diseases.

We examined the ICD-10-GM (icd-code.de, assessed in September 2022) to determine the number of common rare diseases that have a specific diagnosis code. If multiple diseases are grouped under a single ICD code, the code is considered non-specific. For the attribution of ICD codes in chapters see https://www.dimdi.de/static/de/klassifikationen/icd/icd-10-gm/kode-suche/htmlgm2023/.

We compared the subject catalogue for the second written state examination, which outlines the topics covered in the Medical Licensing Regulations and corresponding examination regulations [10], with the dataset obtained from the Orphanet Epidemiology File. Specifically, we focused on Part C, “Disease Profiles“, which contains an annotated and specified catalogue of diseases organized according to ICD-10 codes. Our objective was to determine how many and which of the common rare diseases are explicitly mentioned in the subject catalogue. This could either be through the inclusion of a specific ICD code or a specific annotation associated with the listed ICD codes.

To examine whether there is a significant difference in the frequency of common rare diseases having a specific ICD code or being explicitly mentioned in the subject catalogue based on their prevalence category, we conducted a two sided chi square test. A global significance level of 5% was used to determine statistical significance. We used the Bonferroni correction for multiple testing. Adjusted for two tests the local significance level is a p-value of less than 0,025 for each test.

Additionally, we examined, whether there is a difference in the frequency of having a specific ICD code or being specifically mentioned in the subject catalogue of the common rare diseases depending on their disease chapter in the ICD catalogue. In cases where a particular chapter contained fewer than 5 diseases, we employed a two-sided Fisher’s exact test instead of the chi-square test. P-values are reported only descriptively. For these questions, they are not used to determine statistical significance.

## Results

Among the 454 common rare diseases, the largest proportion (*N* = 106, 23%) fell under ICD chapter XVII “Inborn malformations, deformities and chromosomal abnormalities“.

The second, third and fourth most common disease groups were found in chapters IV (endocrine, nutritional and metabolic diseases), II (neoplasms) and VI (diseases of the nervous system) respectively, comprising 68 (15%), 64 (14%) and 58 (13%) diseases.

Each of the other ICD chapters contained only half as many or less rare diseases.

**Fig. 2:**
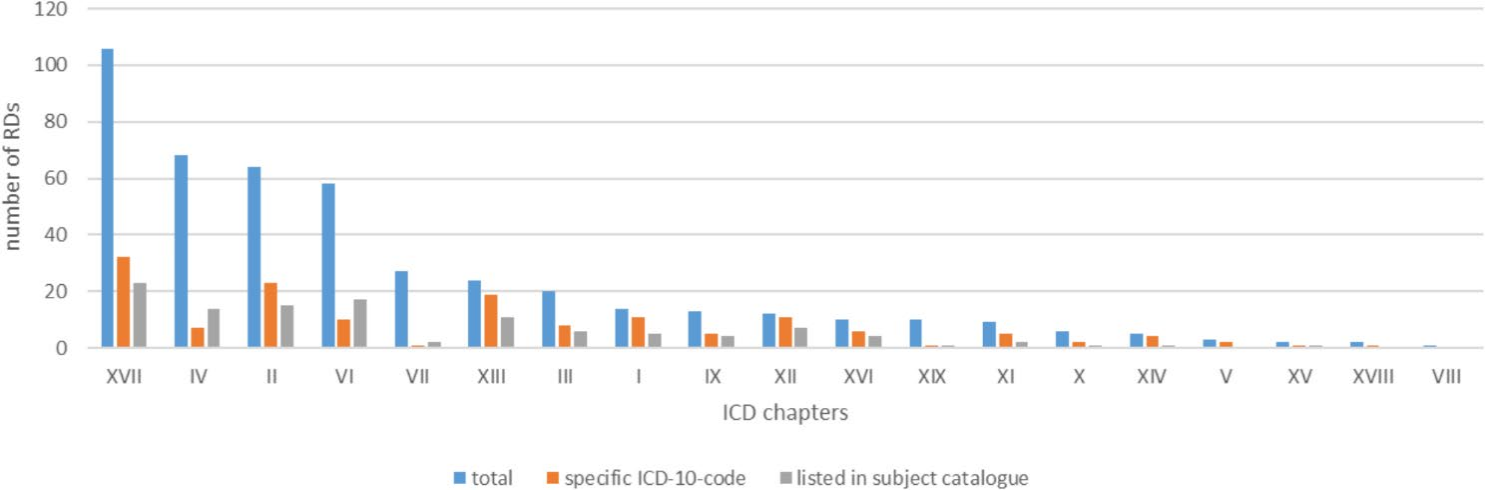
Frequency chart of all 454 rare diseases with prevalence ranges 1–5/10,000 and 1–9/100,000 sorted by ICD chapters

There were no common rare diseases identified within the ICD chapters XX (external factors of morbidity and mortality), XXI (factors, that influence health status and lead to use of the health care system) and XXII (codes for specific purposes) (Fig. 2; Table 2).

**Table 2:** Number of common rare diseases in chapters of the ICD-10-GM

ICD chapter	Total (%)
I	14 (3.1%)
II	64 (14.1%)
III	20 (4.4%)
IV	68 (15%)
V	3 (0.6%)
VI	58 (12.8%)
VII	27 (5.9%)
VIII	1 (0.2%)
IX	13 (2.8%)
X	6 (1.3%)
XI	9 (2%)
XII	12 (2.6%)
XIII	24 (5.3%)
XIV	5 (1.1%)
XV	2 (0.4%)
XVI	10 (2.2%)
XVII	106 (23.3%)
XVIII	2 (0.4%)
XIX	10 (2.2%)
XX	0 (0%)
XXI	0 (0%)
XXII	0 (0%)

### Specific ICD codes

**Fig. 3:**
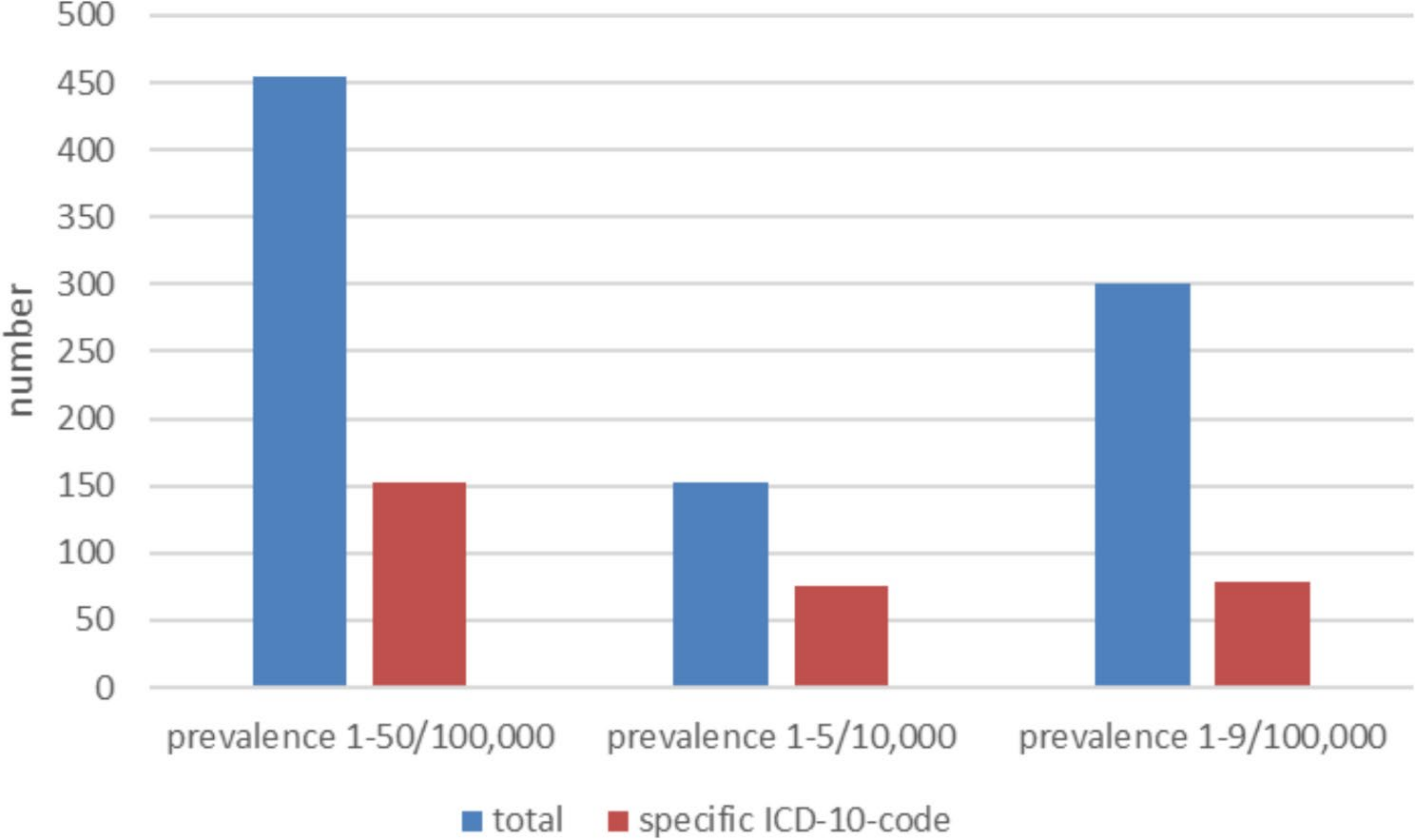
Specific ICD-10-GM codes for all 454 rare diseases, prevalence ranges 1–5/10,000, and 1–9/100,000

Out of the 454 rare diseases analysed, a specific ICD code could be identified for 153 (34%) of them. Specifically, for the most prevalent subset of 153 rare diseases with a prevalence range of 1–5/10,000, a specific ICD code was available for 75 (49%). Furthermore, for the subset of the second most prevalent 301 rare diseases with a prevalence range of 1–9/100,000, a specific ICD code was found for 78 (26%)(Fig. 3, Appendix: Table 6).

A statistically significant difference was observed between the most common rare diseases with a prevalence range of 1–5/10,000 and the second most prevalent rare diseases with a prevalence range of 1–9/100,000 in terms of having a specific ICD-10 code (p-value < 0.001). Rarer diseases were less likely to have a specific ICD code.

### Inclusion in the medical subject catalogue

Out of all rare diseases evaluated, 69 (about 15%) were explicitly mentioned in the subject catalogue. An additional 49 rare diseases had their disease group mentioned in the catalogue, although not the specific diseases themselves. For example, while polycystic kidney disease is mentioned, the autosomal dominant form, which is part of the Orphanet Epidemiology File, is not specifically listed. Similarly, Morbus Fabry is not explicitly mentioned, but the group of sphingolipidoses, to which Morbus Fabry belongs, is included. Taking these additional diseases into account, a total of 118 (26%) of the evaluated rare diseases were covered in the German medical curriculum.

Among the 153 most prevalent rare diseases, 38 (25%) were explicitly mentioned in the subject catalogue. An additional 16 diseases had their group mentioned, meaning that 35% of the most prevalent rare diseases were listed in the catalogue either explicitly or by group.

**Fig. 4:**
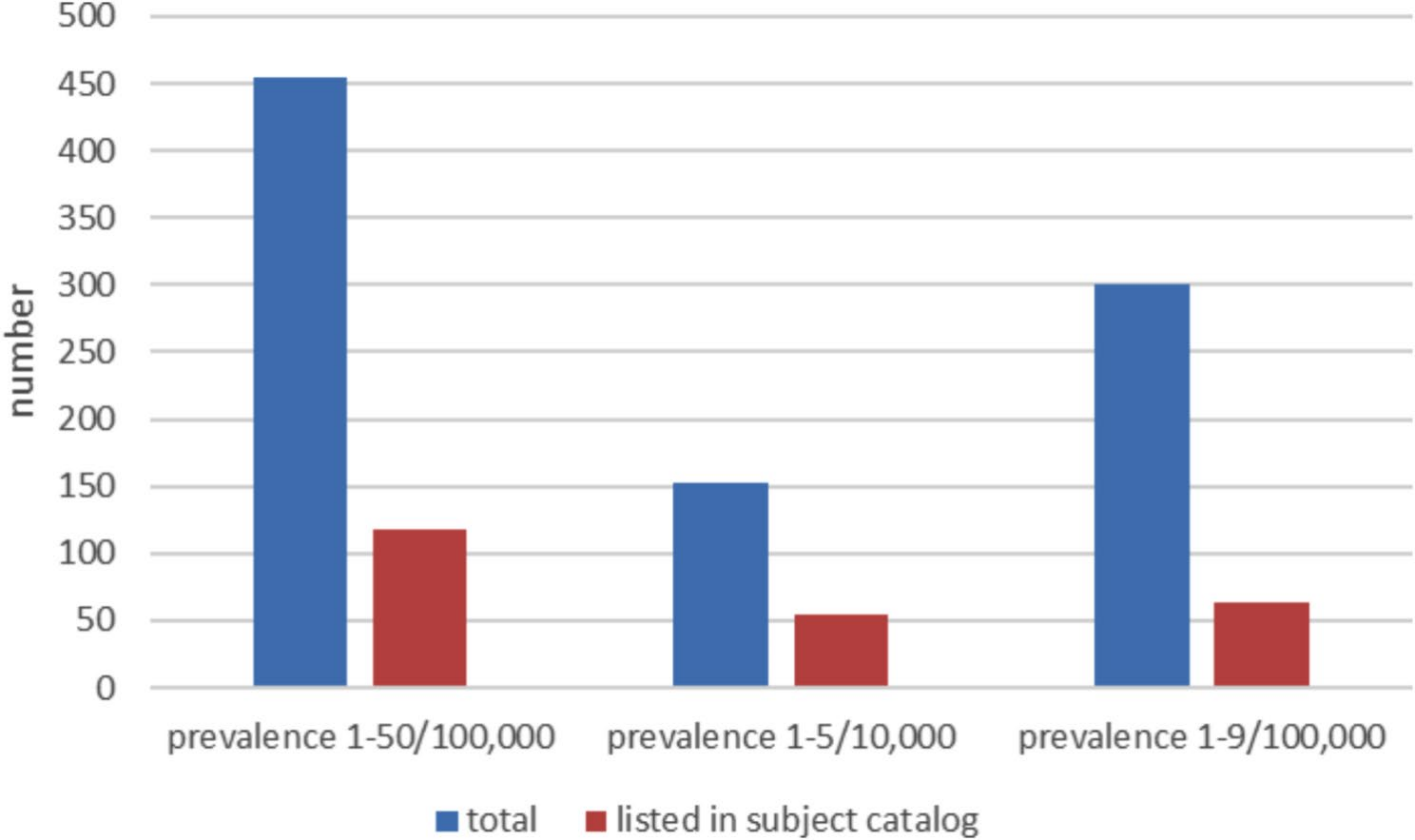
Number of explicitly or implicitly included rare diseases in the subject catalogue of all 454 common rare diseases and prevalence ranges 1–5/10,000, and 1–9/100,000

For the 301 rare diseases with a prevalence range of 1–9/100,000, 31 (11%) were explicitly mentioned in the subject catalogue. With an additional 33 diseases having had their disease group mentioned, a total of 21% of the second most prevalent rare diseases were part of the medical curriculum (Fig. 4, Appendix Table 6).

A statistically significant difference was observed between the most prevalent rare diseases with a prevalence range of 1–5/10,000 and the second most prevalent rare diseases with a prevalence range of 1–9/100,000 in terms of being explicitly mentioned in the subject catalogue (p-value < 0.001). Even when considering the listing of the disease group in the catalogue, the difference remained statistically significant, with a p-value of 0.002. Rarer diseases were less likely to be included in the subject catalogue.

### Differences between disease groups

A comprehensive overview of common rare diseases in each ICD chapter and whether they have a specific ICD-code and are included in the subject catalogue is provided in Table 3.

**Table 3:** Number of common rare diseases that have a specific ICD code and are included in the subjective catalogue in each ICD chapter

ICD chapter	specific ICD-code	*p*-value	Subjectiv catalogue	*p*-value
I	11 (78.6%)	< 0.001	5 (35.7%)	0.454
II	23 (35.9%)	0.726	15 (23.4%)	0.653
III	8 (40%)	0.573	6 (30%)	0.209
IV	7 (10.3%)	< 0.001	14 (20.6%)	0.31
V	2 (66.7%)	0.605	0 (0%)	1
VI	10 (17.2%)	0.003	17 (29.3%)	0.6
VII	1 (3.7%)	< 0.001	2 (7.5%)	< 0.001
VIII	0 (0%)	1	0 (0%)	1
IX	5 (38.5%)	0.728	4 (30.8%)	0.713
X	2 (33.3%)	0.985	1 (16.7%)	0.544
XI	5 (55.6%)	0.191	2 (22.2%)	0.788
XII	11 (91.7%)	< 0.001	7 (58.3%)	0.025
XIII	19 (79.2%)	< 0.001	11 (45.8%)	0.056
XIV	4 (80%)	0.243	1 (20%)	0.74
XV	1 (50%)	1	1 (50%)	0.502
XVI	6 (60%)	0.093	4 (40%)	0.37
XVII	32 (30.2%)	0.481	23 (21.7%)	0.34
XVIII	1 (50%)	1	0 (0%)	0.502
XIX	1 (10%)	0.308	1 (10%)	0.704

A higher proportion of diseases in the chapters I (Specific infectious and parasitic diseases), XII (diseases of the skin and subcutis) and XIII (diseases of the muscular skeletal system and connective tissue diseases) had specific ICD-codes. 79% (*p* < 0,001), 92% (*p* < 0,001) and 79% (*p* < 0,001) of diseases in these chapters had a specific ICD-code, respectively.

In contrast, specific ICD-codes were less frequently found in chapters IV (endocrine, nutritional and metabolic diseases) with 10% (*p* < 0,001), VI (diseases of the nervous system) with 17% (*p* = 0,003) and VII (diseases of the eye and adnexa) with 4% (*p* < 0,001).

Only 7% of the diseases from chapter VII (diseases of the eye and adnexa) are mentioned in the subject catalogue (p value < 0,001), while 58% of the diseases from chapter XII (diseases of the skin and subcutis) are listed in the catalogue (p value 0,025).

No other noticeable differences were observed between disease chapters.

## Discussion

### Coding

49% of the most prevalent rare diseases could be coded specifically whereas only 26% of the second most prevalent rare diseases had a specific ICD code. The difference was statistically significant. We confirmed that the presence of a specific ICD-10 code is dependent on the frequency of the disease, indicating that more common rare diseases are more likely to be assigned specific codes.

However, even among the most prevalent rare diseases, only 49% had a specific code. This highlights the challenge of limited epidemiological data available for rare diseases. To address this issue of inaccurate documentation, the addition of the Alpha-ID to the ICD code when coding rare diseases for billing purposes has been legally mandated in Germany’s inpatient sector since April 1, 2023. The Alpha-ID is a non-classifying diagnostic code that provides a stable identification of medical and/or everyday language terms based on the alphabetical index of the ICD-10-GM. It also includes the Orpha code for rare diseases. The Alpha-ID-SE is updated and extended annually by the German Federal Institute for Drugs and Medical Devices (BfArM). Currently, the Alpha-ID is not yet fully comprehensive and requires annual updates and supplements, limiting its current feasibility. Furthermore, most patients with rare diseases receive outpatient treatment, where specific coding for rare diseases is still not implemented in Germany. Nevertheless, a more comprehensive coding for inpatients represents an important step towards obtaining improved epidemiological data on rare diseases [5, 17]. 

It would be interesting to determine whether the prevalence data from the Orphanet Epidemiology File aligns with the coded cases of rare diseases or whether an update to the Epidemiology File is necessary. For example, Pichon et al. showed in their analyse of the French National Rare Disease Registry that 228 rare diseases have a higher prevalence in France than demonstrated by the Orphanet Epidemiology File [18]. 

### Differences between disease groups

As mentioned, most of the common rare diseases belonged to ICD Chapter XVII, “Inborn malformations, deformities, and chromosomal abnormalities.” This outcome was expected, considering that more than 80% of rare diseases are believed to have a genetic origin [19].

Our study revealed a discrepancy regarding common rare diseases in Chapter IV “Endocrine, nutritional, and metabolic diseases” and Chapter VI “Diseases of the nervous system” of the ICD. Rare diseases belonged 2nd and 4th most frequently to these chapters, respectively, yet diseases in these chapters were less likely to have a specific ICD 10 code compared to rare diseases in other chapters.

Furthermore, the 5th most common rare disease group (Chapter VII - Diseases of the eyes and adnexa) not only demonstrated a lower frequency of having a specific ICD-10 code but was also noticeably less frequently mentioned in the subject catalogue (Fig. 2).

### Medical education subject catalogue

Determining the precise number of diseases represented in the German subject catalogue poses a challenge due to the frequent mention of disease groups rather than individual specific diseases and the catalogue’s inherent incompleteness. Consequently, we were unable to provide the proportion of rare diseases among all mentioned diseases in the catalogue.

Nevertheless, at least 25% of the most prevalent rare diseases were explicitly listed in the catalogue. When considering the disease groups, this percentage rised to over one-third.

The subject catalogue exhibited a lower frequency of rarer diseases. In the prevalence group of 1–9/100,000, which still includes the second most prevalent rare diseases, only 11% were explicitly mentioned, with an additional 10% implicitly described through their corresponding disease groups.

As assessment drives learning, the inclusion of rare diseases in the subject catalogue could enhance medical doctor’s awareness and knowledge about rare diseases [20]. Having encountered these diseases during their medical studies could enhance the probability of recognizing it in patients. Like Johann Wolfgang von Goethe (1749–1832, German poet and scientist) stated in a letter to F. von Müller in 1819, “You only catch sight of, what you already know and understand.” A medical doctor’s knowledge on the existence of these rare diseases could therefore contribute to accurate diagnoses. Assuming, that approximately 80% of individuals affected by a rare disease have one of roughly 150 most prevalent rare diseases [15], it means that in Germany, with an estimated 5 million rare disease patients, doctors would have encountered the diseases of at least 4 million individuals during their study times if another 115 of the most prevalent rare diseases were explicitly mentioned in the subject catalogue and taught in medical schools accordingly. Transferring Pareto’s Principle to the field of rare diseases, medical students only need knowledge on 1.9 to 2.5% of all rare diseases (150/6000–8000) to be able to recognize 80% of rare disease patients, making the cost-benefit-analysis even more efficient than the original Pareto Principle.

It is not realistic to include all common rare diseases in medical education. Using rare diseases to teach pathophysiologic principles and teaching students how to handle a state of not knowing could be very beneficial, though. It could increase the general awareness on rare diseases as well as familiarize students with supporting structures like centres for rare diseases, undiagnosed disease programs and knowledgebases for rare diseases like the Orphanet classification and encyclopedia of rare diseases. Hopefully, such measures would help address some common problems of patients with rare diseases, like the diagnostic odyssey, lack of information provided at the time of diagnosis, insufficient coordination of care, and low or non-existent access to medication and therapies due to poor knowledge and lacking research and clinical trials [13, 21, 22]. 

### Limitations

We did not consider the ultra-rare diseases in our analysis. However, it can be presumed that the observed correlation between disease frequency, its mention in the subject catalogue, and the presence of a specific ICD code also applies to these ultra-rare diseases. Namely, that these ultra-rare diseases are significantly less likely to have a specific ICD code and are also mentioned less frequently in the catalogue. For example, 153 unique codes are given to the 454 common rare diseases with a prevalence of 1/2,000–1/100,000. Aymé et al. showed that only 355 of the 6,954 clinical entities listed by Orphanet have a unique specific code in the ICD-10 WHO version 2015. Another 162 diseases can be specifically mapped to a set of ICD 10 codes [4]. That leaves about 6,500 diseases with 202 specific codes, which corresponds to about 3% of the rarer diseases having a specific code.

The Orphanet Epidemiology File does not include all rare diseases. It contains only 5,000 rare diseases out of approximately 8,000 known rare diseases. Gaps still exist in this database. There are 3,700 diseases with usable prevalence information. However, we assume that the more common diseases have a more comprehensive representation in this file, as there is more epidemiological data available for common rare diseases compared to ultra-rare diseases.

By way of example, we focused our analysis on the situation in Germany, regarding both the specific German version of the ICD-10 as well as the German subject catalogue for the medical state examinations. Representation of rare diseases in medical curricula may vary due to differences in the national medical curricula.

## Conclusions

As assessment drives learning, efforts should be made to include at least the most prevalent rare diseases in medical education. Regional variations in prevalence should be considered as well as the addition of teaching modules on rare and undiagnosed diseases in general. This could enhance students’ and physicians’ awareness of rare diseases.

## Electronic supplementary material

Below is the link to the electronic supplementary material.

Supplementary Material 1

## Data Availability

All data supporting the findings of this study are available within the paper and its supplementary information files.

## References

[1] Union R, der E. Empfehlung des Rates vom 8. Juni 2009 für eine Maßnahme im Bereich seltener Krankheiten [Internet]. Amtsblatt der Europäischen Union Nr. C 1512009. 2009 [cited 2023 May 15]. https://eur-lex.europa.eu/LexUriServ/LexUriServ.do?uri=CELEX:32009H0703%2802%29:DE:HTML

[2] Eidt D, Frank M, Reimann A, Wagner TOF, Mittendorf T, Schulenburg J-MG, von der. Maßnahmen zur Verbesserung der gesundheitlichen Situation von Menschen mit seltenen Erkrankungen in Deutschland - Studie im Auftrag des Bundesministeriums für Gesundheit [Internet]. Bundesministerium für Gesundheit Referat Öffentlichkeitsarbeit; 2009 Jun. https://www.namse.de/fileadmin/user_upload/downloads/BMG_Forschungsbericht_Seltene_Krankheiten.pdf

[3] Schieppati A, Henter J-I, Daina E, Aperia A. Why rare diseases are an important medical and social issue. Lancet. 2008;371:2039–41. 18555915 10.1016/S0140-6736(08)60872-7

[4] Aymé S, Bellet B, Rath A. Rare diseases in ICD11: making rare diseases visible in health information systems through appropriate coding. Orphanet J Rare Dis. 2015;10:35. 25887186 10.1186/s13023-015-0251-8 PMC4377208

[5] Martin T, Rommel K, Thomas C, Eymann J, Kretschmer T, Berner R, et al. Seltene Erkrankungen in den Daten Sichtbar machen – kodierung. Bundesgesundheitsblatt - Gesundheitsforschung - Gesundheitsschutz. 2022;65:1133–42. 36239768 10.1007/s00103-022-03598-9 PMC9636302

[6] Nikendei C, Weyrich P, Jünger J, Schrauth M. Medical education in Germany. Méd Teach. 2009;31:591–600. 19811144 10.1080/01421590902833010

[7] Georgantopoulou C. Medical education in Greece. Méd Teach. 2009;31:13–7. 19253151 10.1080/01421590802331453

[8] Cate OT. Medical education in the Netherlands. Méd Teach. 2007;29:752–7. 18236272 10.1080/01421590701724741

[9] Segouin C, Jouquan J, Hodges B, Bréchat P, David S, Maillard D, et al. Country report: medical education in France. Méd Educ. 2007;41:295–301. 17316215 10.1111/j.1365-2929.2007.02690.x

[10] IMPP-Gegenstandskatalog. (IMPP-GK2) für den schriftlichen Teil des zweiten Abschnitts der Ärztlichen Prüfung [Internet]. [cited 2023 Jun 29]. https://www.impp.de/files/PDF/Gegenstandskataloge/Medizin/gk2-2021-Auflage05_1.pdf

[11] Innern ED. des. Informationen über die eidgenössische Prüfung Humanmedizin [Internet]. 2024 [cited 2024 May 22]. https://www.bag.admin.ch/bag/de/home/berufe-im-gesundheitswesen/medizinalberufe/eidgenoessische-pruefungen-universitaerer-medizinalberufe/eidgenoessische-pruefung-in-humanmedizin.html

[12] PROFILESMED.CH [Internet]. [cited 2024 May 22]. https://www.profilesmed.ch/

[13] de Vries E, Fransen L, Aker M, van den, Meijboom BR. Preventing gatekeeping delays in the diagnosis of rare diseases. Br J Gen Pr. 2018;68:145–6. 10.3399/bjgp18X695225 PMC5819975 29472225

[14] Powell T, Sammut-Bonnici T. Wiley Encyclopedia Manage. 2017;1–2.

[15] Wakap SN, Lambert DM, Olry A, Rodwell C, Gueydan C, Lanneau V, et al. Estimating cumulative point prevalence of rare diseases: analysis of the Orphanet database. Eur J Hum Genet. 2020;28:165–73. 31527858 10.1038/s41431-019-0508-0 PMC6974615

[16] Orphanet Epidemiology File [Internet]. 2022 [cited 2022 Sep 14]. https://www.orphadata.com/epidemiology/

[17] Dennler U. Die Kodierung Seltener Erkrankungen. KU Gesundheitsmanagement. 2023;85–8.

[18] Pichon T, Messiaen C, Soussand L, Angin C, Sandrin A, Elarouci N, et al. Overview of patients’ cohorts in the French National rare disease registry. Orphanet J Rare Dis. 2023;18:176. 37400917 10.1186/s13023-023-02725-2 PMC10318625

[19] Vairo FPe, Kemppainen JL, Vitek CRR, Whalen DA, Kolbert KJ, Sikkink KJ, et al. Implementation of genomic medicine for rare disease in a tertiary healthcare system: Mayo Clinic Program for Rare and Undiagnosed diseases (PRaUD). J Transl Med. 2023;21:410. 37353797 10.1186/s12967-023-04183-7 PMC10288779

[20] McLachlan JC. The relationship between assessment and learning. Méd Educ. 2006;40:716–7. 16869912 10.1111/j.1365-2929.2006.02518.x

[21] Limb NS, Sen. and A. Experiences of rare diseases: an insight from patients and families. Rare Dis UK. 2010.

[22] Molster C, Urwin D, Pietro LD, Fookes M, Petrie D, van der Laan S, et al. Survey of healthcare experiences of Australian adults living with rare diseases. Orphanet J Rare Dis. 2016;11:30. 27012247 10.1186/s13023-016-0409-z PMC4806449

