# Supplementary material for: Pareto-principle in rare disease education: assessing the representation of common rare diseases in medical education and coding systems
Source: Orphanet J Rare Dis. 2024 Sep 12;19:340. doi: 10.1186/s13023-024-03347-y (PMC11396778; doi:10.1186/s13023-024-03347-y)
Supplement: Supplementary file 1 — Supplementary Material 1 [file 13023_2024_3347_MOESM1_ESM.docx]

# Appendix

Table 4: 30 diseases excluded from analysis due to multiple prevalence data of which the prevalence range did not fall within the target range in Germany, Europe or worldwide

| Orphacode | Rare disease | Prevalence orphanet | Location | Prevalence that leaded to exclusion |
| --- | --- | --- | --- | --- |
| 54 | X-linked recessive ocular albinism | 1-9 / 100,000 | United States | Europe 1-9/1,000,000 |
| 56 | Alkaptonuria | 1-9 / 100,000 | Slovakia | Europe 1-9/1,000,000 |
| 100 | Ataxia-teleangiectasia | 1-9 / 100,000 | Italy | Europe 1-9/1,000,000 |
| 207 | Crouzon syndrome | 1-9 / 100,000 | Canada | Europe 1-9/1,000,000 |
| 256 | Early-onset generalized limb-onset dystonia | 1-9 / 100,000 | United States | Europe 1-9/1,000,000 |
| 313 | Lamellar ichthyosis | 1-9 / 100,000 | Norway | Europe 1-9/1,000,000 |
| 550 | MELAS | 1-9 / 100,000 | Finland | Europe 1-9/1,000,000 |
| 579 | Mucopolysaccharidosis type 1 | 1-5 / 10,000 | Norway | Europe 1-9/1,000,000 |
| 580 | Mucopolysaccharidosis type 2 | 1-9 / 100,000 | Denmark | Europe 1-9/1,000,000 |
| 581 | Mucopolysaccharidosis type 3 | 1-9 / 100,000 | Sweden | Europe 1-9/1,000,000 |
| 583 | Mucopolysaccharidosis type 6 | 1-9 / 100,000 | Sweden | Europe 1-9/1,000,000 |
| 611 | Inclusion body myositis | 1-9 / 100,000 | Australia | Europe 1-9/1,000,000 |
| 767 | Polyarteritis nodosa | 1-9 / 100,000 | Europa | Germany 1-9/1,000,000 |
| 813 | Silver-Russell syndrome | 1-9 / 100,000 | United Kingdom | Europe 1-9/1,000,000 |
| 816 | Sjögren-Larsson syndrome | 1-5 / 10,000 | Taiwan, Province of China | Europe 1-9/1,000,000 |
| 821 | Sotos syndrome | 1-9 / 100,000 | Weltweit | Europe 1-9/1,000,000 |
| 902 | Werner syndrome | 1-9 / 100,000 | Japan | Europe 1-9/1,000,000 |
| 1572 | Common variable immunodeficiency | 1-9 / 100,000 | Europa | Germany 1-9/1,000,000 |
| 2573 | Moyamoya disease | 1-9 / 100,000 | Japan | Europe 1-9/1,000,000 |
| 3378 | Trisomy 13 | 1-5 / 10,000 | Japan | Europe 1-9/1,000,000 |
| 3380 | Trisomy 18 | 1-5 / 10,000 | Japan | Europe 1-9/1,000,000 |
| 3386 | American trypanosomiasis | 1-9 / 100,000 | Spain | Germany <1/1,000,000 |
| 3453 | Autoimmune polyendocrinopathy type 1 | 1-9 / 100,000 | Finland | Europe 1-9/1,000,000 |
| 35909 | Combined deficiency of factor V and factor VIII | 1-9 / 100,000 | Iran, Islamic Republic of | Europe 1-9/1,000,000 |
| 79276 | Acute intermittent porphyria | 1-5 / 10,000 | Sweden | Europe 1-9/1,000,000 |
| 79278 | Autosomal erythropoietic protoporphyria | 1-9 / 100,000 | Netherlands | Europe 1-9/1,000,000 |
| 79430 | Hermansky-Pudlak syndrome | 1-5 / 10,000 | Puerto rico | worldwide 1-9/1,000,000 |
| 79473 | Porphyria variegata | 1-9 / 100,000 | Switzerland | Europe 1-9/1,000,000 |
| 98758 | Spinocerebellar ataxia type 6 | 1-9 / 100,000 | Japan | worldwide1-9/1,000,000 |
| 98879 | Hemophilia B | 1-9 / 100,000 | Europa | Germany 1-9/1,000,000 |

Table 5: 18 diseases excluded due to describing complications or therapy consequences rather than primary disease entities

| Orphacode | Rare disease |
| --- | --- |
| 39812 | Graft versus host disease |
| 70475 | Radiation proctitis |
| 70568 | Post-transplant lymphoproliferative disease |
| 90052 | Recurrent hepatitis C virus induced liver disease in liver transplant recipients |
| 90073 | Hepatitis B reinfection following liver transplantation |
| 90080 | Scarring in glaucoma filtration surgical procedures |
| 90081 | AIDS wasting syndrome |
| 91127 | Adenovirus infection in immunocompromised patients |
| 94059 | Uremic pruritus |
| 137698 | Cytomegalovirus disease in patients with impaired cell mediated immunity deemed at risk |
| 217067 | Pouchitis |
| 217080 | Pulmonary fungal infections in patients deemed at risk |
| 231080 | Highgrade dysplasia in patients with Barrett’s esophagus |
| 238621 | Ileal pouch anal anastomosis related faecal incontinence |
| 263352 | Postcardiotomy right ventricular failure |
| 268316 | Complication in hemodialysis |
| 306644 | Complication after organ transplantation |
| 391655 | Off-periods in Parkinson disease not responding to oral treatment |

Table 6: results for all included common rare diseases

| Orphacode | Rare disease | Prevalence range | Specific ICD-10 | Mentioned in subject catalogue |
| --- | --- | --- | --- | --- |
| 5 | Long chain 3-hydroxyacyl-CoA dehydrogenase deficiency | 1-9 / 100,000 | no | no |
| 6 | 3-methylcrotonyl-CoA carboxylase deficiency | 1-9 / 100,000 | no | no |
| 8 | 47,XYY syndrome | 1-5 / 10,000 | yes | no |
| 15 | Achondroplasia | 1-9 / 100,000 | no | no |
| 16 | Blue cone monochromatism | 1-9 / 100,000 | no | no |
| 23 | Argininosuccinic aciduria | 1-9 / 100,000 | no | no |
| 33 | Isovaleric acidemia | 1-9 / 100,000 | no | no |
| 41 | Dyschromatosis symmetrica hereditaria | 1-9 / 100,000 | no | no |
| 42 | Medium chain acyl-CoA dehydrogenase deficiency | 1-9 / 100,000 | no | no |
| 48 | Congenital bilateral absence of vas deferens | 1-5 / 10,000 | no | no |
| 51 | Aicardi-Goutières syndrome | 1-5 / 10,000 | no | no |
| 52 | Alagille syndrome | 1-9 / 100,000 | no | no |
| 53 | Albers-Schönberg osteopetrosis | 1-9 / 100,000 | no | no |
| 60 | Alpha-1-antitrypsin deficiency | 1-5 / 10,000 | no | no |
| 65 | AmauroLeber congenital amaurosissis congenita Leber | 1-9 / 100,000 | no | no |
| 68 | Amoebiasis due to free-living amoebae | 1-9 / 100,000 | yes | no |
| 70 | Proximal spinal muscular atrophy | 1-9 / 100,000 | yes | no |
| 72 | Angelman syndrome | 1-9 / 100,000 | no | no |
| 81 | Antisynthetase syndrome | 1-9 / 100,000 | no | no |
| 82 | Hereditary thrombophilia due to congenital antithrombin deficiency | 1-5 / 10,000 | no | no |
| 87 | Apert syndrome | 1-9 / 100,000 | no | no |
| 95 | Friedreich ataxia | 1-9 / 100,000 | no | no |
| 102 | Multiple system atrophy | 1-9 / 100,000 | yes | no |
| 104 | Leber hereditary optic neuropathy | 1-9 / 100,000 | no | no |
| 107 | BOR syndrome | 1-9 / 100,000 | no | no |
| 110 | Bardet Biedl syndrome | 1-9 / 100,000 | no | no |
| 116 | Beckwith-Wiedemann syndrome | 1-5 / 10,000 | no | no |
| 117 | Behçet disease | 1-9 / 100,000 | yes | no |
| 130 | Brugada syndrome | 1-5 / 10,000 | no | no |
| 131 | Budd-Chiari syndrome | 1-9 / 100,000 | yes | no |
| 132 | Hereditary butyrylcholinesterase deficiency | 1-9 / 100,000 | no | no |
| 136 | Cerebral autosomal dominant arteriopathy-subcortical infarcts-leukoencephalopathy | 1-9 / 100,000 | no | no |
| 138 | CHARGE syndrome | 1-9 / 100,000 | no | no |
| 145 | Hereditary breast and/or ovarian cancer syndrome | 1-5 / 10,000 | no | no |
| 150 | Nasopharyngeal carcinoma | 1-9 / 100,000 | yes | no |
| 154 | Familial isolated dilated cardiomyopathy | 1-5 / 10,000 | no | no |
| 157 | Carnitine palmitoyltransferase II deficiency | 1-9 / 100,000 | no | no |
| 171 | Primary sclerosing cholangitis | 1-9 / 100,000 | yes | no |
| 177 | Rhizomelic chondrodysplasia punctata | 1-9 / 100,000 | no | no |
| 180 | Choroideremia | 1-9 / 100,000 | no | no |
| 183 | Eosinophilic granulomatosis with polyangiitis | 1-9 / 100,000 | yes | no |
| 186 | Primary biliary cholangitis | 1-5 / 10,000 | yes | no |
| 189 | Hidrotic ectodermal dysplasia | 1-9 / 100,000 | no | no |
| 192 | Coffin-Lowry syndrome | 1-9 / 100,000 | no | no |
| 195 | Cat-eye syndrome | 1-9 / 100,000 | no | no |
| 205 | Crigler-Najjar syndrome | 1-9 / 100,000 | yes | no |
| 212 | Cystathioninuria | 1-9 / 100,000 | no | no |
| 213 | Cystinosis | 1-9 / 100,000 | no | no |
| 214 | Cystinuria | 1-9 / 100,000 | no | yes |
| 217 | Isolated Dandy-Walker malformation | 1-9 / 100,000 | yes | no |
| 218 | Darier disease | 1-9 / 100,000 | no | no |
| 221 | Dermatomyositis | 1-9 / 100,000 | yes | yes |
| 232 | Sickle cell anemia | 1-5 / 10,000 | yes | no |
| 233 | Duane retraction syndrome | 1-5 / 10,000 | no | no |
| 243 | 46,XX gonadal dysgenesis | 1-9 / 100,000 | no | no |
| 244 | Primary ciliary dyskinesia | 1-5 / 10,000 | no | no |
| 267 | Calpain-3-related limb-girdle muscular dystrophy R1 | 1-9 / 100,000 | no | mentioned as group |
| 269 | Facioscapulohumeral dystrophy | 1-9 / 100,000 | no | mentioned as group |
| 270 | Oculopharyngeal muscular dystrophy | 1-9 / 100,000 | no | mentioned as group |
| 273 | Steinert myotonic dystrophy | 1-9 / 100,000 | no | mentioned as group |
| 276 | T-B+ severe combined immunodeficiency due to gamma chain deficiency | 1-9 / 100,000 | no | no |
| 280 | Wolf-Hirschhorn syndrome | 1-9 / 100,000 | no | no |
| 285 | Hypermobile Ehlers-Danlos syndrome | 1-5 / 10,000 | no | no |
| 287 | Classical Ehlers-Danlos syndrome | 1-9 / 100,000 | no | no |
| 288 | Hereditary elliptocytosis | 1-5 / 10,000 | yes | no |
| 294 | Fetal cytomegalovirus syndrome | 1-5 / 10,000 | yes | no |
| 296 | Ollier disease | 1-9 / 100,000 | no | no |
| 308 | Progressive myoclonic epilepsy type 1 | 1-9 / 100,000 | no | no |
| 321 | Multiple osteochondromas | 1-9 / 100,000 | yes | no |
| 324 | Fabry disease | 1-5 / 10,000 | no | mentioned as group |
| 340 | Hemorrhagic fever-renal syndrome | 1-5 / 10,000 | no | no |
| 342 | Familial Mediterranean fever | 1-5 / 10,000 | no | no |
| 355 | Gaucher disease | 1-9 / 100,000 | no | mentioned as group |
| 358 | Gitelman syndrome | 1-9 / 100,000 | no | no |
| 360 | Glioblastoma | 1-9 / 100,000 | no | no |
| 365 | Glycogen storage disease due to acid maltase deficiency | 1-9 / 100,000 | no | mentioned as group |
| 377 | Gorlin syndrome | 1-9 / 100,000 | no | no |
| 389 | Langerhans cell histiocytosis | 1-9 / 100,000 | no | yes |
| 391 | Classic Hodgkin lymphoma | 1-5 / 10,000 | yes | yes |
| 393 | 46,XX testicular difference of sex development | 1-9 / 100,000 | no | no |
| 394 | Homocystinuria due to cystathionine beta-synthase deficiency | 1-9 / 100,000 | no | yes |
| 396 | Chronic hiccup | 1-9 / 100,000 | no | no |
| 397 | Giant cell arteritis | 1-9 / 100,000 | yes | yes |
| 399 | Huntington disease | 1-5 / 10,000 | yes | yes |
| 412 | Dysbetalipoproteinemia | 1-9 / 100,000 | no | no |
| 414 | Gyrate atrophy of choroid and retina | 1-9 / 100,000 | no | no |
| 422 | Idiopathic/heritable pulmonary arterial hypertension | 1-9 / 100,000 | yes | no |
| 429 | Hypochondroplasia | 1-9 / 100,000 | no | no |
| 447 | Paroxysmal nocturnal hemoglobinuria | 1-9 / 100,000 | yes | no |
| 461 | Recessive X-linked ichthyosis | 1-5 / 10,000 | no | mentioned as group |
| 469 | Hereditary fructose intolerance | 1-9 / 100,000 | no | yes |
| 480 | Kearns-Sayre syndrome | 1-9 / 100,000 | no | no |
| 481 | Kennedy disease | 1-9 / 100,000 | no | no |
| 487 | Krabbe disease | 1-9 / 100,000 | no | mentioned as group |
| 521 | Chronic myeloid leukemia | 1-9 / 100,000 | yes | no |
| 524 | Li-Fraumeni syndrome | 1-9 / 100,000 | no | no |
| 536 | Systemic lupus erythematosus | 1-5 / 10,000 | yes | yes |
| 543 | Burkitt lymphoma | 1-5 / 10,000 | yes | no |
| 545 | Follicular lymphoma | 1-5 / 10,000 | yes | yes |
| 552 | MODY | 1-5 / 10,000 | no | no |
| 558 | Marfan syndrome | 1-5 / 10,000 | yes | yes |
| 567 | 22q11.2 deletion syndrome | 1-5 / 10,000 | no | no |
| 569 | Familial or sporadic hemiplegic migraine | 1-5 / 10,000 | no | no |
| 582 | Mucopolysaccharidosis type 4 | 1-5 / 10,000 | no | no |
| 586 | Cystic fibrosis | 1-5 / 10,000 | yes | yes |
| 589 | Myasthenia gravis | 1-5 / 10,000 | yes | yes |
| 602 | GNE myopathy | 1-9 / 100,000 | no | mentioned as group |
| 606 | Proximal myotonic myopathy | 1-9 / 100,000 | no | mentioned as group |
| 609 | Tibial muscular dystrophy | 1-9 / 100,000 | no | mentioned as group |
| 614 | Thomsen and Becker disease | 1-9 / 100,000 | no | mentioned as group |
| 616 | Medulloblastoma | 1-9 / 100,000 | no | no |
| 626 | Large congenital melanocytic nevus | 1-9 / 100,000 | no | no |
| 628 | Diastrophic dysplasia | 1-9 / 100,000 | yes | no |
| 631 | Non-acquired isolated growth hormone deficiency | 1-5 / 10,000 | no | no |
| 635 | Neuroblastoma | 1-5 / 10,000 | no | yes |
| 636 | Neurofibromatosis type 1 | 1-5 / 10,000 | no | mentioned as group |
| 637 | Full NF2-related schwannomatosis | 1-9 / 100,000 | no | mentioned as group |
| 640 | Hereditary neuropathy with liability to pressure palsies | 1-9 / 100,000 | no | no |
| 641 | Multifocal motor neuropathy | 1-9 / 100,000 | no | no |
| 644 | NARP syndrome | 1-9 / 100,000 | no | no |
| 646 | Niemann-Pick disease type C | 1-9 / 100,000 | no | mentioned as group |
| 648 | Noonan syndrome | 1-5 / 10,000 | no | no |
| 652 | Multiple endocrine neoplasia type 1 | 1-9 / 100,000 | no | no |
| 653 | Multiple endocrine neoplasia type 2 | 1-9 / 100,000 | no | no |
| 654 | Multiple endocrine neoplasia type 2 | 1-9 / 100,000 | no | yes |
| 660 | Nephroblastoma | 1-5 / 10,000 | yes | yes |
| 664 | Ornithine transcarbamylase deficiency | 1-9 / 100,000 | no | no |
| 666 | Osteogenesis imperfecta | 1-5 / 10,000 | yes | yes |
| 668 | Osteosarcoma | 1-9 / 100,000 | no | no |
| 673 | Malaria | 1-9 / 100,000 | yes | yes |
| 681 | Hypokalemic periodic paralysis | 1-9 / 100,000 | no | no |
| 683 | Progressive supranuclear palsy | 1-5 / 10,000 | yes | no |
| 700 | Alopecia totalis | 1-5 / 10,000 | yes | no |
| 701 | Alopecia universalis | 1-5 / 10,000 | yes | no |
| 703 | Bullous pemphigoid | 1-5 / 10,000 | yes | mentioned as group |
| 704 | Pemphigus vulgaris | 1-9 / 100,000 | yes | mentioned as group |
| 705 | Pendred syndrome | 1-9 / 100,000 | no | no |
| 716 | Phenylketonuria | 1-5 / 10,000 | yes | yes |
| 718 | Isolated Pierre Robin syndrome | 1-9 / 100,000 | no | no |
| 727 | Microscopic polyangiitis | 1-9 / 100,000 | yes | no |
| 729 | Polycythemia vera | 1-5 / 10,000 | yes | yes |
| 730 | Autosomal dominant polycystic kidney disease | 1-5 / 10,000 | yes | mentioned as group |
| 732 | Polymyositis | 1-9 / 100,000 | yes | yes |
| 733 | Familial adenomatous polyposis | 1-9 / 100,000 | no | yes |
| 739 | Prader-Willi syndrome | 1-9 / 100,000 | no | no |
| 746 | Mitochondrial trifunctional protein deficiency | 1-9 / 100,000 | no | no |
| 750 | Pseudoachondroplasia | 1-9 / 100,000 | no | no |
| 758 | Pseudoxanthoma elasticum | 1-9 / 100,000 | no | no |
| 759 | Precocious puberty | 1-5 / 10,000 | no | no |
| 761 | Immunoglobulin A vasculitis | 1-9 / 100,000 | no | no |
| 766 | Polyarteritis nodosa | 1-9 / 100,000 | no | no |
| 774 | Hereditary hemorrhagic telangiectasia | 1-5 / 10,000 | yes | no |
| 778 | Rett syndrome | 1-5 / 10,000 | yes | no |
| 783 | Rubinstein-Taybi syndrome | 1-9 / 100,000 | no | no |
| 791 | Retinitis pigmentosa | 1-5 / 10,000 | no | yes |
| 792 | X-linked retinoschisis | 1-9 / 100,000 | no | no |
| 794 | Saethre-Chotzen syndrome | 1-9 / 100,000 | no | no |
| 797 | Sarcoidosis | 1-5 / 10,000 | yes | yes |
| 803 | Amyotrophic lateral sclerosis | 1-9 / 100,000 | no | no |
| 805 | Tuberous sclerosis complex | 1-9 / 100,000 | yes | no |
| 809 | Mixed connective tissue disease | 1-9 / 100,000 | no | no |
| 819 | Smith-Magenis syndrome | 1-9 / 100,000 | no | no |
| 822 | Hereditary spherocytosis | 1-5 / 10,000 | yes | yes |
| 824 | Primary myelofibrosis | 1-9 / 100,000 | no | yes |
| 827 | Stargardt disease | 1-5 / 10,000 | no | no |
| 828 | Stickler syndrome | 1-9 / 100,000 | no | no |
| 829 | Adult-onset Still disease | 1-9 / 100,000 | yes | no |
| 846 | Alpha-thalassemia | 1-5 / 10,000 | yes | no |
| 853 | Fetal and neonatal alloimmune thrombocytopenia | 1-5 / 10,000 | no | no |
| 854 | Primitive portal vein thrombosis | 1-9 / 100,000 | no | yes |
| 861 | Treacher-Collins syndrome | 1-9 / 100,000 | yes | no |
| 870 | Down syndrome | 1-5 / 10,000 | yes | yes |
| 881 | Turner syndrome | 1-5 / 10,000 | yes | yes |
| 886 | Usher syndrome | 1-9 / 100,000 | no | no |
| 888 | Van der Woude syndrome | 1-9 / 100,000 | no | no |
| 889 | Cutaneous small vessel vasculitis | 1-9 / 100,000 | no | no |
| 892 | Von Hippel-Lindau disease | 1-9 / 100,000 | no | no |
| 900 | Granulomatosis with polyangiitis | 1-9 / 100,000 | yes | no |
| 903 | Von Willebrand disease | 1-5 / 10,000 | yes | yes |
| 905 | Wilson disease | 1-9 / 100,000 | no | no |
| 908 | Fragile X syndrome | 1-5 / 10,000 | yes | yes |
| 909 | Cerebrotendinous xanthomatosis | 1-9 / 100,000 | no | no |
| 910 | Xeroderma pigmentosum | 1-9 / 100,000 | yes | no |
| 913 | Zollinger-Ellison syndrome | 1-9 / 100,000 | no | no |
| 926 | Acatalasemia | 1-9 / 100,000 | no | no |
| 930 | Idiopathic achalasia | 1-9 / 100,000 | yes | no |
| 963 | Acromegaly | 1-9 / 100,000 | no | yes |
| 976 | Adenine phosphoribosyltransferase deficiency | 1-9 / 100,000 | no | no |
| 1020 | Early-onset autosomal dominant Alzheimer disease | 1-9 / 100,000 | no | mentioned as group |
| 1114 | Aplasia cutis congenita | 1-5 / 10,000 | no | no |
| 1177 | Early-onset cerebellar ataxia with retained tendon reflexes | 1-9 / 100,000 | no | no |
| 1199 | Esophageal atresia | 1-5 / 10,000 | yes | yes |
| 1203 | Duodenal atresia | 1-9 / 100,000 | no | yes |
| 1209 | Tricuspid atresia | 1-9 / 100,000 | no | no |
| 1243 | Best vitelliform macular dystrophy | 1-9 / 100,000 | no | no |
| 1330 | Partial atrioventricular septal defect | 1-5 / 10,000 | no | mentioned as group |
| 1332 | Medullary thyroid carcinoma | 1-9 / 100,000 | no | no |
| 1478 | Interatrial communication | 1-9 / 100,000 | no | mentioned as group |
| 1552 | Currarino syndrome | 1-9 / 100,000 | no | no |
| 1598 | Monosomy 18q | 1-9 / 100,000 | no | no |
| 1646 | Partial chromosome Y deletion | 1-5 / 10,000 | no | no |
| 1656 | Dermatitis herpetiformis | 1-5 / 10,000 | yes | yes |
| 1872 | Cone rod dystrophy | 1-9 / 100,000 | no | no |
| 1873 | Jalili syndrome | 1-9 / 100,000 | no | no |
| 1880 | Ebstein malformation of the tricuspid valve | 1-9 / 100,000 | yes | mentioned as group |
| 1896 | EEC syndrome | 1-9 / 100,000 | no | no |
| 1928 | Congenital lobar emphysema | 1-9 / 100,000 | no | no |
| 1941 | Juvenile absence epilepsy | 1-9 / 100,000 | no | no |
| 2017 | Sternal cleft | 1-9 / 100,000 | no | no |
| 2019 | Femur-fibula-ulna complex | 1-9 / 100,000 | no | no |
| 2023 | Undifferentiated pleomorphic sarcoma | 1-9 / 100,000 | no | no |
| 2032 | Idiopathic pulmonary fibrosis | 1-5 / 10,000 | no | no |
| 2070 | Eosinophilic gastroenteritis | 1-9 / 100,000 | no | no |
| 2073 | Narcolepsy type 1 | 1-5 / 10,000 | no | no |
| 2116 | Hartnup disease | 1-9 / 100,000 | no | no |
| 2134 | Atypical hemolytic uremic syndrome | 1-9 / 100,000 | no | no |
| 2137 | Autoimmune hepatitis | 1-5 / 10,000 | yes | yes |
| 2138 | 46,XX ovotesticular difference of sex development | 1-9 / 100,000 | no | no |
| 2157 | Histidinemia | 1-9 / 100,000 | no | no |
| 2314 | Autosomal dominant hyper-IgE syndrome due to STAT3 deficiency | 1-9 / 100,000 | no | no |
| 2322 | Kabuki syndrome | 1-9 / 100,000 | no | no |
| 2337 | Diffuse palmoplantar keratoderma, Bothnian type | 1-9 / 100,000 | no | no |
| 2345 | Isolated Klippel-Feil syndrome | 1-9 / 100,000 | yes | no |
| 2368 | Gastroschisis | 1-5 / 10,000 | yes | yes |
| 2382 | Lennox-Gastaut syndrome | 1-5 / 10,000 | no | no |
| 2398 | Multiple symmetric lipomatosis | 1-9 / 100,000 | no | no |
| 2440 | Isolated split hand-split foot malformation | 1-9 / 100,000 | no | no |
| 2481 | Neurocutaneous melanocytosis | 1-9 / 100,000 | no | no |
| 2614 | Nail-patella syndrome | 1-9 / 100,000 | no | no |
| 2764 | Osteochondritis dissecans | 1-5 / 10,000 | yes | yes |
| 2828 | Young-onset Parkinson disease | 1-5 / 10,000 | no | mentioned as group |
| 2901 | Neuralgic amyotrophy | 1-5 / 10,000 | yes | no |
| 2903 | Familial spontaneous pneumothorax | 1-5 / 10,000 | no | no |
| 2911 | Poland syndrome | 1-9 / 100,000 | no | no |
| 2924 | Isolated polycystic liver disease | 1-9 / 100,000 | no | no |
| 2932 | Chronic inflammatory demyelinating polyneuropathy | 1-9 / 100,000 | no | no |
| 2965 | Prolaktinoma | 1-5 / 10,000 | no | no |
| 3002 | Immune thrombocytopenia | 1-5 / 10,000 | no | no |
| 3109 | Mayer-Rokitansky-Küster-Hauser syndrome | 1-5 / 10,000 | no | no |
| 3129 | Sarcosinemia | 1-9 / 100,000 | no | no |
| 3165 | Eosinophilic fasciitis | 1-9 / 100,000 | yes | no |
| 3193 | Supravalvular aortic stenosis | 1-5 / 10,000 | yes | mentioned as group |
| 3205 | Sturge-Weber syndrome | 1-9 / 100,000 | no | no |
| 3286 | Catecholaminergic polymorphic ventricular tachycardia | 1-5 / 10,000 | no | no |
| 3287 | Takayasu arteritis | 1-9 / 100,000 | yes | no |
| 3318 | Essential thrombocythemia | 1-5 / 10,000 | yes | no |
| 3375 | Trisomy X | 1-5 / 10,000 | yes | no |
| 3389 | Tuberculosis | 1-5 / 10,000 | yes | yes |
| 3392 | Tularemia | 1-9 / 100,000 | yes | yes |
| 3426 | Double outlet right ventricle | 1-5 / 10,000 | yes | no |
| 3440 | Waardenburg syndrome | 1-9 / 100,000 | no | no |
| 3449 | Weill-Marchesani syndrome | 1-9 / 100,000 | no | no |
| 3451 | Infantile spasms syndrome | 1-9 / 100,000 | no | no |
| 3465 | Worster-Drought syndrome | 1-9 / 100,000 | no | mentioned as group |
| 26790 | Pseudomyxoma peritonei | 1-9 / 100,000 | no | no |
| 26793 | Very long chain acyl-CoA dehydrogenase deficiency | 1-9 / 100,000 | no | no |
| 29073 | Multiple myeloma | 1-5 / 10,000 | yes | yes |
| 29207 | Reactive arthritis | 1-9 / 100,000 | yes | yes |
| 30391 | Isolated biliary atresia | 1-9 / 100,000 | yes | yes |
| 31112 | Dermatofibrosarcoma protuberans | 1-5 / 10,000 | no | no |
| 33208 | Idiopathic hypersomnia | 1-5 / 10,000 | yes | no |
| 33226 | Waldenström macroglobulinemia | 1-9 / 100,000 | yes | no |
| 33276 | Kaposi sarcoma | 1-9 / 100,000 | yes | yes |
| 33475 | Meningococcal meningitis | 1-9 / 100,000 | yes | no |
| 34515 | FKRP-related limb-girdle muscular dystrophy R9 | 1-9 / 100,000 | no | mentioned as group |
| 35122 | Congenital sucrase-isomaltase deficiency | 1-5 / 10,000 | no | no |
| 35689 | Primary lateral sclerosis | 1-9 / 100,000 | no | no |
| 36234 | Bacterial toxic-shock syndrome | 1-9 / 100,000 | yes | yes |
| 36258 | Buerger disease | 1-5 / 10,000 | yes | yes |
| 37202 | Interstitial cystitis | 1-5 / 10,000 | yes | no |
| 39044 | Uveal melanoma | 1-9 / 100,000 | no | yes |
| 42062 | Iminoglycinuria | 1-9 / 100,000 | no | no |
| 43393 | Lambert-Eaton myasthenic syndrome | 1-9 / 100,000 | yes | no |
| 44890 | Gastrointestinal stromal tumor | 1-5 / 10,000 | no | no |
| 45453 | Incessant infant ventricular tachycardia | 1-9 / 100,000 | no | no |
| 46486 | Mucous membrane pemphigoid | 1-9 / 100,000 | yes | mentioned as group |
| 46724 | Cerebral arteriovenous malformation | 1-9 / 100,000 | yes | no |
| 49042 | Dentinogenesis imperfecta | 1-5 / 10,000 | no | no |
| 49382 | Achromatopsia | 1-9 / 100,000 | no | no |
| 50251 | Pleural mesothelioma | 1-9 / 100,000 | yes | yes |
| 50839 | Cat-scratch disease | 1-9 / 100,000 | yes | yes |
| 52416 | Mantle cell lymphoma | 1-9 / 100,000 | no | no |
| 52417 | MALT lymphoma | 1-9 / 100,000 | yes | no |
| 54370 | Primary membranoproliferative glomerulonephritis | 1-5 / 10,000 | yes | no |
| 54595 | Craniopharyngioma | 1-9 / 100,000 | no | yes |
| 55880 | Chondrosarcoma | 1-9 / 100,000 | no | no |
| 57145 | SUNCT syndrome | 1-9 / 100,000 | no | no |
| 58017 | Classic hairy cell leukemia | 1-9 / 100,000 | yes | no |
| 60015 | Enlarged parietal foramina | 1-9 / 100,000 | no | no |
| 60032 | Recurrent respiratory papillomatosis | 1-9 / 100,000 | no | no |
| 60039 | Pudendal neuralgia | 1-5 / 10,000 | no | no |
| 66627 | Tenosynovial giant cell tumor | 1-5 / 10,000 | yes | no |
| 67038 | B-cell chronic lymphocytic leukemia | 1-5 / 10,000 | yes | no |
| 67043 | Amoebic keratitis | 1-9 / 100,000 | no | no |
| 70476 | Vernal keratoconjunctivitis | 1-5 / 10,000 | no | no |
| 70567 | Cholangiocarcinoma | 1-9 / 100,000 | yes | yes |
| 70573 | Small cell lung cancer | 1-5 / 10,000 | no | no |
| 70578 | Adult acute respiratory distress syndrome | 1-5 / 10,000 | yes | no |
| 70587 | Infant acute respiratory distress syndrome | 1-5 / 10,000 | yes | yes |
| 70588 | Meconium aspiration syndrome | 1-9 / 100,000 | yes | no |
| 70589 | Bronchopulmonary dysplasia | 1-5 / 10,000 | yes | yes |
| 71211 | Neuromyelitis optica spectrum disorder | 1-9 / 100,000 | yes | yes |
| 75377 | Central areolar choroidal dystrophy | 1-9 / 100,000 | no | no |
| 75564 | Acquired idiopathic sideroblastic anemia | 1-9 / 100,000 | no | no |
| 79086 | Acquired generalized lipodystrophy | 1-9 / 100,000 | no | no |
| 79126 | Acute interstitial pneumonia | 1-9 / 100,000 | no | no |
| 79140 | Cutaneous neuroendocrine carcinoma | 1-9 / 100,000 | no | no |
| 79241 | Biotinidase deficiency | 1-9 / 100,000 | no | no |
| 79264 | Juvenile neuronal ceroid lipofuscinosis | 1-9 / 100,000 | yes | mentioned as group |
| 79432 | Oculocutaneous albinism type 2 | 1-9 / 100,000 | no | no |
| 79435 | Oculocutaneous albinism type 4 | 1-9 / 100,000 | no | no |
| 79481 | Pemphigus foliaceus | 1-9 / 100,000 | yes | mentioned as group |
| 79501 | Punctate palmoplantar keratoderma type 1 | 1-9 / 100,000 | no | no |
| 83313 | Boutonneuse fever | 1-5 / 10,000 | no | no |
| 83317 | Scrub typhus | 1-9 / 100,000 | yes | no |
| 83461 | Congenital primary aphakia | 1-5 / 10,000 | yes | no |
| 83463 | Mikrotia | 1-5 / 10,000 | yes | no |
| 83465 | Narcolepsy type 2 | 1-5 / 10,000 | no | no |
| 85138 | Addison disease | 1-5 / 10,000 | no | yes |
| 85408 | Rheumatoid factor-negative polyarticular juvenile idiopathic arthritis | 1-9 / 100,000 | yes | mentioned as group |
| 85410 | Oligoarticular juvenile idiopathic arthritis | 1-5 / 10,000 | yes | mentioned as group |
| 85414 | Systemic-onset juvenile idiopathic arthritis | 1-9 / 100,000 | yes | mentioned as group |
| 85435 | Rheumatoid factor-positive polyarticular juvenile idiopathic arthritis | 1-9 / 100,000 | no | mentioned as group |
| 85436 | Psoriasis-related juvenile idiopathic arthritis | 1-9 / 100,000 | no | mentioned as group |
| 85438 | Enthesitis-related juvenile idiopathic arthritis | 1-9 / 100,000 | no | mentioned as group |
| 85443 | AL amyloidosis | 1-5 / 10,000 | no | no |
| 85446 | Wild type ABeta2M amyloidosis | 1-9 / 100,000 | no | no |
| 85447 | ATTRV30M amyloidosis | 1-9 / 100,000 | no | no |
| 86814 | Benign adult familial myoclonic epilepsy | 1-9 / 100,000 | no | no |
| 86867 | Nodal marginal zone B-cell lymphoma | 1-9 / 100,000 | no | no |
| 86870 | Blastic plasmacytoid dendritic cell neoplasm | 1-5 / 10,000 | yes | no |
| 86875 | Adult T-cell leukemia/lymphoma | 1-9 / 100,000 | yes | no |
| 87503 | Mal de Meleda | 1-9 / 100,000 | no | no |
| 88629 | Tritanopia | 1-9 / 100,000 | no | no |
| 88661 | Amelogenesis imperfecta | 1-9 / 100,000 | no | no |
| 89936 | X-linked hypophosphatemia | 1-9 / 100,000 | yes | no |
| 90033 | Autoimmune hemolytic anemia, warm type | 1-9 / 100,000 | no | mentioned as group |
| 90050 | Retinopathy of prematurity | 1-5 / 10,000 | yes | yes |
| 90051 | Sepsis in premature infants | 1-5 / 10,000 | no | mentioned as group |
| 90056 | Moderate and severe traumatic brain injury | 1-5 / 10,000 | no | mentioned as group |
| 90058 | Spinal cord injury | 1-5 / 10,000 | yes | yes |
| 90059 | Sudden sensorineural hearing loss | 1-5 / 10,000 | no | no |
| 90060 | Diffuse alveolar hemorrhage | 1-9 / 100,000 | no | no |
| 90062 | Acute liver failure | 1-5 / 10,000 | no | no |
| 90064 | Acute peripheral arterial occlusion | 1-5 / 10,000 | yes | yes |
| 90065 | Acquired aneurysmal subarachnoid hemorrhage | 1-5 / 10,000 | no | mentioned as group |
| 90066 | Pneumonia caused by Pseudomonas aeruginosa infection | 1-5 / 10,000 | yes | no |
| 90068 | Cocaine intoxication | 1-9 / 100,000 | yes | no |
| 90076 | Partial deep dermal and full thickness burns | 1-5 / 10,000 | yes | mentioned as group |
| 90289 | Localized scleroderma | 1-9 / 100,000 | yes | yes |
| 90291 | Systemic sclerosis | 1-5 / 10,000 | yes | yes |
| 90794 | Classic congenital adrenal hyperplasia due to 21-hydroxylase deficiency | 1-9 / 100,000 | yes | no |
| 91138 | Cryoglobulinemic vasculitis | 1-9 / 100,000 | no | yes |
| 91349 | Non-functioning pituitary adenoma | 1-5 / 10,000 | no | no |
| 93110 | Posterior urethral valve | 1-9 / 100,000 | yes | no |
| 93256 | Fragile X-associated tremor/ataxia syndrome | 1-9 / 100,000 | no | no |
| 93321 | Radial hemimelia | 1-9 / 100,000 | yes | no |
| 93323 | Fibular hemimelia | 1-9 / 100,000 | yes | no |
| 93402 | Syndactyly type 1 | 1-5 / 10,000 | no | no |
| 94058 | Neovascular glaucoma | 1-5 / 10,000 | no | no |
| 95712 | Thyroid ectopia | 1-5 / 10,000 | no | no |
| 95713 | Athyreosis | 1-9 / 100,000 | no | no |
| 95715 | Congenital hypothyroidism due to transplacental passage of TSH-binding inhibitory antibodies | 1-9 / 100,000 | no | no |
| 95716 | Familial thyroid dyshormonogenesis | 1-9 / 100,000 | no | no |
| 95719 | Thyroid hemiagenesis | 1-5 / 10,000 | no | no |
| 95720 | Thyroid hypoplasia | 1-9 / 100,000 | no | no |
| 96253 | Cushing disease | 1-9 / 100,000 | no | yes |
| 96263 | 48,XXXY syndrome | 1-9 / 100,000 | no | no |
| 97230 | Solar urticaria | 1-9 / 100,000 | yes | no |
| 97292 | Cardiogenic shock | 1-5 / 10,000 | yes | no |
| 98673 | Autosomal dominant optic atrophy, classic form | 1-9 / 100,000 | no | no |
| 98755 | Spinocerebellar ataxia type 1 | 1-9 / 100,000 | no | no |
| 98756 | Spinocerebellar ataxia type 2 | 1-9 / 100,000 | no | no |
| 98757 | Spinocerebellar ataxia type 3 | 1-9 / 100,000 | no | no |
| 98838 | Primary mediastinal large B-cell lymphoma | 1-9 / 100,000 | no | no |
| 98841 | Anaplastic large cell lymphoma | 1-9 / 100,000 | yes | no |
| 98848 | Indolent systemic mastocytosis | 1-5 / 10,000 | no | no |
| 98849 | Systemic mastocytosis with associated hematologic neoplasm | 1-9 / 100,000 | no | no |
| 98878 | Hemophilia A | 1-9 / 100,000 | yes | yes |
| 98895 | Becker muscular dystrophy | 1-9 / 100,000 | no | mentioned as group |
| 98896 | Duchenne muscular dystrophy | 1-9 / 100,000 | no | mentioned as group |
| 98916 | Acute inflammatory demyelinating polyradiculoneuropathy | 1-9 / 100,000 | no | no |
| 98969 | Macular corneal dystrophy | 1-9 / 100,000 | no | no |
| 98973 | Posterior polymorphous corneal dystrophy | 1-9 / 100,000 | no | no |
| 98976 | Congenital glaucoma | 1-9 / 100,000 | no | no |
| 98977 | Juvenile glaucoma | 1-9 / 100,000 | no | no |
| 99772 | Cleft velum | 1-5 / 10,000 | yes | mentioned as group |
| 99828 | Dengue fever | 1-9 / 100,000 | yes | no |
| 99953 | Charcot-Marie-Tooth disease type 4G | 1-5 / 10,000 | no | no |
| 99976 | Adenocarcinoma of the esophagus | 1-9 / 100,000 | no | no |
| 99977 | Squamous cell carcinoma of the esophagus | 1-9 / 100,000 | no | no |
| 99978 | Klatskin tumor | 1-9 / 100,000 | no | no |
| 99981 | Apnea of prematurity | 1-9 / 100,000 | no | no |
| 100070 | Progressive non-fluent aphasia | 1-9 / 100,000 | no | no |
| 100075 | Neuroendocrine tumor of stomach | 1-9 / 100,000 | no | no |
| 101016 | Romano-Ward syndrome | 1-5 / 10,000 | no | no |
| 101081 | Charcot-Marie-Tooth disease type 1A | 1-5 / 10,000 | no | no |
| 101082 | Charcot-Marie-Tooth disease type 1B | 1-9 / 100,000 | no | no |
| 101330 | Porphyria cutanea tarda | 1-9 / 100,000 | yes | no |
| 137577 | Neonatal hypoxic and ischemic brain injury | 1-9 / 100,000 | yes | no |
| 137583 | Vulvar intraepithelial neoplasia | 1-5 / 10,000 | yes | no |
| 137596 | Neurotrophic keratopathy | 1-5 / 10,000 | no | no |
| 137599 | Herpes simplex virus stromal keratitis | 1-5 / 10,000 | no | no |
| 137686 | Asherman syndrome | 1-5 / 10,000 | yes | no |
| 137914 | Choanal atresia | 1-9 / 100,000 | no | yes |
| 139417 | Acute transverse myelitis | 1-9 / 100,000 | yes | no |
| 140949 | Low-flow priapism | 1-9 / 100,000 | yes | mentioned as group |
| 157798 | Serrated polyposis syndrome | 1-5 / 10,000 | no | no |
| 163934 | Atopic keratoconjunctivitis | 1-5 / 10,000 | no | no |
| 168782 | Childhood disintegrative disorder | 1-9 / 100,000 | no | no |
| 168811 | Malignant peritoneal mesothelioma | 1-9 / 100,000 | yes | no |
| 171436 | Typical nemaline myopathy | 1-9 / 100,000 | no | mentioned as group |
| 171673 | Limbal stem cell deficiency | 1-5 / 10,000 | no | no |
| 178029 | Central diabetes insipidus | 1-9 / 100,000 | yes | yes |
| 178320 | Acute lung injury | 1-5 / 10,000 | no | no |
| 180242 | Malignant tumor of fallopian tubes | 1-9 / 100,000 | yes | no |
| 199302 | Isolated cleft lip | 1-5 / 10,000 | yes | mentioned as group |
| 199306 | Cleft lip/palate | 1-5 / 10,000 | yes | mentioned as group |
| 209989 | Non-papillary transitional cell carcinoma of the bladder | 1-5 / 10,000 | no | no |
| 217064 | 5-fluorouracil poisoning | 1-9 / 100,000 | no | no |
| 221061 | Familial cerebral cavernous malformation | 1-5 / 10,000 | no | no |
| 228113 | Anal fistula | 1-5 / 10,000 | yes | yes |
| 238468 | Hypohidrotic ectodermal dysplasia | 1-9 / 100,000 | no | no |
| 238624 | Idiopathic intracranial hypertension | 1-5 / 10,000 | yes | no |
| 247234 | Sporadic adult-onset ataxia of unknown etiology | 1-9 / 100,000 | no | no |
| 247525 | Citrullinemia type I | 1-9 / 100,000 | no | no |
| 250923 | Isolated aniridia | 1-9 / 100,000 | yes | no |
| 251076 | 8p23.1 duplication syndrome | 1-9 / 100,000 | no | no |
| 252164 | Benign schwannoma | 1-9 / 100,000 | no | no |
| 261197 | Proximal 16p11.2 microdeletion syndrome | 1-5 / 10,000 | no | no |
| 263432 | Nevus of Ito | 1-9 / 100,000 | no | no |
| 275555 | Preeclampsia | 1-5 / 10,000 | yes | yes |
| 275761 | Lysosomal acid lipase deficiency | 1-9 / 100,000 | no | no |
| 275864 | Behavioral variant of frontotemporal dementia | 1-9 / 100,000 | no | no |
| 280200 | Microform holoprosencephaly | 1-9 / 100,000 | no | no |
| 281090 | Syndromic recessive X-linked ichthyosis | 1-9 / 100,000 | no | mentioned as group |
| 289157 | Hypocalcemic vitamin D-dependent rickets | 1-5 / 10,000 | no | no |
| 289390 | Primary Sjögren syndrome | 1-5 / 10,000 | yes | no |
| 300557 | Carcinoma of the ampulla of Vater | 1-9 / 100,000 | yes | no |
| 330001 | Wild type ATTR amyloidosis | 1-5 / 10,000 | no | no |
| 330015 | Lead poisoning | 1-9 / 100,000 | yes | no |
| 330021 | Mercury poisoning | 1-9 / 100,000 | yes | no |
| 330064 | Chronic actinic dermatitis | 1-5 / 10,000 | yes | no |
| 331206 | Severe combined immunodeficiency due to complete RAG1/2 deficiency | 1-9 / 100,000 | no | no |
| 352731 | Oculocutaneous albinism type 1 | 1-9 / 100,000 | no | no |
| 391673 | Necrotizing enterocolitis | 1-5 / 10,000 | yes | yes |
| 402823 | Hepatitis delta | 1-5 / 10,000 | yes | no |
| 404448 | ADNP syndrome | 1-9 / 100,000 | no | no |
| 411527 | Central retinal vein occlusion | 1-5 / 10,000 | no | no |
| 411703 | Pulmonary non-tuberculous mycobacterial infection | 1-9 / 100,000 | yes | no |
| 439167 | Placental insufficiency | 1-5 / 10,000 | no | no |
| 449266 | Pleural empyema | 1-5 / 10,000 | no | yes |
| 449285 | Snakebite envenomation | 1-9 / 100,000 | yes | no |
| 565782 | Methotrexate toxicity | 1-9 / 100,000 | no | no |
